# Synergistic Effects of 9,10-Dihydro-9-oxa-10-phosphaphenanthrene-10-oxide-Based Derivative and Modified Sepiolite on Flame-Retarded Poly (Ethylene Oxide)–Poly (Butylene Adipate-Co-Terephthalate) Composites

**DOI:** 10.3390/polym16010045

**Published:** 2023-12-22

**Authors:** Weijiang Huang, Chunyun Tu, Qin Tian, Kui Wang, Chunlin Yang, Chao Ma, Xiaolu Xu, Wei Yan

**Affiliations:** 1 College of Materials Science and Engineering, Guiyang University, Guiyang 550005, China; yidapa@sina.cn (C.T.); mabtianqin@126.com (Q.T.); gyxywkui@163.com (K.W.); ycl770309@163.com (C.Y.); chaomagyu@126.com (C.M.); gyxyxxl@126.com (X.X.); 2 National Engineering Research Center for Compounding and Modification of Polymer Materials, Guiyang 550014, China

**Keywords:** poly (ethylene oxide), poly (butylene adipate-co-terephthalate), DOPO-based flame retardant, sepiolite, synergistic effect

## Abstract

A 9,10-dihydro-9-oxa-10-phosphaphenanthrene-10-oxide (DOPO)-based derivative (PN-DOPO) combined with aluminium phosphates-coated sepiolite (Sep@AlPO_4_) was used to improve the flame retardance, thermal stability and mechanical performances of poly (ethylene oxide) (PEO)/poly (butylene adipate-co-terephthalate) (PBAT) blends. The synergistic effects of PN-DOPO and Sep@AlPO_4_ on flame-retarded PEO/PBAT composites were systematically discussed. Results indicated that introducing 5 wt% Sep@AlPO_4_ with 10 wt% PN-DOPO into PEO/PBAT achieved a V-1 rating for the UL-94 test and increased the limiting oxygen index value to 23.7%. Moreover, the peak heat release rate (p-HRR), average HRR and total heat release values of PEO/PBAT/PN10%/Sep5% composites decreased by 35.6%, 11.0% and 23.0% compared with those of PEO/PBAT, respectively. Thermogravimetric analysis (TGA) results confirmed that PN-DOPO/Sep@AlPO_4_ enhanced the initial thermal stability and char yield of PEO/PBAT matrix, and TGA/Fourier transform infrared spectrometry results revealed that the composites exhibited the characteristic absorption peaks of phosphorous-containing groups and an increase in gas-phase volatiles during thermal degradation. The morphological structures of the residues indicated that PN-DOPO and Sep@AlPO_4_ mixtures produced a more dense and continuous char layer on the composite surface during burning. Rheological behaviour revealed that higher complex viscosity and modulus values of PEO/PBAT/PN-DOPO/Sep@AlPO_4_ sample could also promote the crosslinking network structure of condensed phases during combustion. Furthermore, the PEO/PBAT/PN-DOPO/Sep@AlPO_4_ composites exhibited superior elongation at break and flexural performance than the PEO/PBAT system. All results demonstrated that the PEO/PBAT system modified with PN-DOPO/Sep@AlPO_4_ showed remarkable flame retardance, and improved thermal stability and mechanical properties, indicating its potential application in areas requiring fire safety.

## 1. Introduction

Poly (ethylene oxide) (PEO) is a typical matrix for solid polymer electrolyte electrolytes (SPEs) because lithium salts show good solubility in PEO, thereby promoting the generation of charge carriers for conductive ions [1,2]. Additionally, PEO can be used for moulding and synthesising water-soluble films owing to its low toxicity and good biological adhesion. However, neat PEO has low thermal stability and poor mechanical strength and rheological properties at room temperature [3,4,5]. Therefore, blending PEO with other polymers effectively improves the shortcomings of PEO-based matrices.

Recently, PEO-biopolymer blends can be considered as one of the research directions to improve the performance of the matrix, based on the high requirements for sustainable, green, renewable and biodegradable materials [6]. Poly (butylene adipate-co-terephthalate) (PBAT), which is a fully biodegradable polyester, is frequently combined with other polymers owing to its excellent elongation at break, good processing properties and hydrophilicity [7,8]. Thus, PBAT is considered a promising and practical counterpart for blending with PEO. Ye et al. [6] prepared PBAT/poly (lactic acid) (PLA)-based nanocomposites by melt-blending PLA/PBAT with PEO/graphene nanosheets (GNP). Incorporating a small amount of PEO into GNP could enhance its dispersion in the matrix, which considerably enhances the thermal behaviour and mechanical performances of nanocomposites.

However, both PEO and PBAT are safety hazards as they are highly flammable [9,10,11,12]. They have a low limiting oxygen index (LOI, less than 21%) and exhibit serious melt dripping during combustion. PEO-based SPEs are usually operated at higher temperatures, i.e., higher than 60 °C. Thus, PEO-based blends should exhibit high flame retardance and good thermal properties [13,14,15]. Therefore, to enhance the security of the matrix and reduce the fire risk under high-temperature conditions, high-performance PEO-based composites with excellent flame retardancy must be developed. However, few studies have focused on designing PEO-based blends with superior fire resistance and rheological properties [16].

Among the numerous flame retardants (FRs), 9,10-dihydro-9-oxa-10-phosphaphenanthrene-10-oxide (DOPO)-based compounds have attracted considerable attention owing to their outstanding fire resistance and low toxicity when DOPO is blended with various polymers such as polyesters, epoxies, polyamides and cellulose [17,18,19,20]. In previous studies, a DOPO-based FR (PN-DOPO) with excellent flame retardancy when combined with polyamide, epoxy resin, polylactic acid and polyolefins was prepared and verified [21,22,23,24]. To further enhance the fire resistance of DOPO-based FRs, synergistic agents can be introduced into the composites, such as montmorillonite, sepiolite, graphite nanosheets, carbon nanotube and halloysite [25,26,27]. Among them, sepiolite, which is a natural silicate clay material with a unique nanofibrous structure, is a potential FR or reinforcing agent. However, when only sepiolite is used as an FR, it does not exhibit satisfying flame retardancy [28,29]. Thus, an organic–inorganic hybrid FR was prepared by coating the sepiolite surface with aluminium phosphate (Sep@AlPO_4_) via a simple precipitation strategy based on our previous reports [30]. Adding 20 wt% Sep@AlPO_4_ into epoxy resin, the LOI value of composites increased to 30.1% and achieved a V-0 rating in a UL-94 test. Moreover, aluminium phosphate-coated sepiolite significantly enhanced the thermal property and mechanical performance of the matrix. As far as we know, there are few studies on the flame-retardant effects between phosphorus-containing FR and modified sepiolite for PEO/PBAT blends. Therefore, this work aims to enhance the fire resistance, mechanical performance and thermal properties of PEO/PBAT by introducing self-made PN-DOPO and Sep@AlPO_4_.

PEO/PBAT/PN-DOPO/Sep@AlPO_4_ composites were prepared by melt blending with PEO/PBAT as the matrix in this study, and PN-DOPO was made in our laboratory as a flame retardant and aluminium phosphate-coated sepiolite as a synergist. The morphology structure and chemical composition of the residues after combustion were characterised. Furthermore, the synergistic flame retardance, thermal behaviour and mechanical performance of a combination of PN-DOPO and Sep@AlPO_4_ on the PEO/PBAT blend were systematically discussed.

## 2. Materials and Methods

### 2.1. Materials

The PEO (M_w_: 100,000 g/mol) was brought from Macklin Biochemical Co., Ltd., Shanghai, China. The melt flow index is 8.0 g/10 min (at 100 °C, 2.16 kg) with a density of 0.93 g/cm^3^ at 25 °C. The melting temperature of PEO is 90 °C. The PBAT resin (M_n_: 24,400 g/mol) was from Jinhui Zhaolong High-Tech Co., Ltd., Taiyuan, China. The melt flow index is 5 g/10 min (at 185 °C) with a density of 1.25 g/cm^3^. The FR (PN-DOPO) and synergist (aluminium phosphate-coated sepiolite, Sep@AlPO_4_) were synthesised in the laboratory according to the protocols described in previous reports [23,30,31].

### 2.2. Preparation of PEO/PBAT/PN-DOPO/Sep@AlPO_4_ Composites

PEO, PBAT, Sep@AlPO_4_ and PN-DOPO were dried at 85 °C for 5 h. PEO/PBAT/PN-DOPO/Sep@AlPO_4_ composites were melt-mixed at 150 °C through a torque rheometer (Haake PolyLab OS, Thermo Fisher Scientific Co., Ltd., Karlsruhe, Germany). The compositions of the samples are listed in Table 1. The compounds were first heat-pressed (16 MPa) at 150 °C for 12 min. Then, they were made into sheets of appropriate size and thickness at 25 °C for 20 min with 16 MPa through a plate vulcaniser (ZHY-W-1, Chengde Testing Machine Factory, Chengde, China).

### 2.3. Characterisation

The UL-94 rating was measured with the sample size of 130.0 × 13.0 × 3.2 mm^3^ according to ASTM D3801 [32]. The LOI values were tested using a JF-3 oxygen index tester (Nanjing Jiangning Instrument, Nanjing, China), where the sample dimension was 130.0 × 6.5 × 3.2 mm^3^. The cone calorimeter tests were conducted using a cone calorimeter (Fire Testing Technology, East Grinstead, UK) based on standard ISO 5660-1 [33], where the sample size was 100.0 × 100.0 × 6.0 mm^3^ under an external heat flux of 50 kW/m^2^. Each sample was tested at least thrice, and the repeatability of the test results should be within ±10%.

Thermal decomposition behaviours of PEO/PBAT and flame-retarded PEO/PBAT composites were measured using a thermogravimetric analyser (TGA, TG 219 F3, Netzsch Instruments, Selbu, Germany) in a N_2_ atmosphere. The specimen (about 5–10 mg) was heated at 30–800 °C with a heating rate of 10 °C/min.

Rheological behaviours of the composites were obtained using a rheometer (HAAKE MARSII, Thermo Fisher Co., Ltd., Karlsruhe, Germany) with parallel-plate geometry (25 mm diameter and 1 mm thickness). Frequency sweeping was performed from 0.01 to 100 rad/s with 1% strain, and the tests were performed at 150 °C.

The morphological structure of the residues after the cone calorimeter tests was determined using scanning electron microscopy (SEM, Quanta 250, FEI Instruments, Waltham, MA, USA) under the voltage of 20 kV. Energy-dispersive spectrometry (EDS, Energy 350, Oxford Instruments, Oxford, UK) was used to determine the composition of the char layer. The EDS tests were performed at the voltage of 30 kV.

The gases released from TGA were investigated using a Nicolet iS50 Fourier transform infrared (FTIR) spectrometer (Nicolet iS50, Thermo Fisher, Waltham, MA, USA). Furthermore, the TGA–FTIR analysis was conducted using thermogravimetry coupled with FTIR. The FTIR spectra were collected over 16 scans for each sample at a wave number interval between 500 and 4000 cm^−1^.

Mechanical properties of the samples were performed using a CMT6104 universal polymer testing machine (MTS Systems Corporation, Shanghai, China) with a constant rate of 50.0 mm/min. The flexural tests were performed at a constant rate of 2.0 mm/min. Each sample was tested at least at least five times, and the repeatability of the test results should be within ±5%.

## 3. Results and Discussion

### 3.1. Flame Retardance of PEO/PBAT/PN-DOPO/Sep@AlPO_4_ Composites

The flame retardance of the PEO/PBAT and PEO/PBAT/PN-DOPO/Sep@AlPO4 composites were measured through UL-94 vertical burning and an LOI test, and the corresponding experimental results are presented in Table 2. Owing to the intrinsic dripping characteristics and low LOI values of PLA and PBAT, the PEO/PBAT sample exhibited severe dripping during combustion, and the LOI value was approximately 20.2%. The recording flame times of the samples exceeded 30 s and had no flame-retardant rating. When 15 wt% PN-DOPO was introduced into the PEO/PBAT blend, a UL-94 V-2 rating was attained and the LOI value increased to 22.6%. At only a Sep@AlPO_4_ loading of 15 wt%, the LOI of PEO/PBAT/Sep15% was 21.8%, and the composites also attained a V-2 rating. The dripping phenomenon was still evident.

The combination of PN-DOPO and Sep@AlPO_4_ was added into the PEO/PBAT blends to further reform the flame-retardant efficiency. A PN-DOPO/Sep@AlPO_4_ loading of 15 wt% was also used in the PEO/PBAT blends. When 3 wt% and 5 wt% Sep@AlPO_4_ displaced the same amount of PN-DOPO, respectively, PEO/PBAT/PN-DOPO/Sep@AlPO_4_ composites reached the V-1 rating and exhibited anti-dripping performance. Furthermore, the LOI value of PEO/PBAT/PN10%/Sep5% sample was increased to 23.7%. The results indicated that a notable synergistic flame-retardant effect occurred between Sep@AlPO_4_ and PN-DOPO, and the combinations could obviously reform the flame retardancy of PEO/PBAT blends.

### 3.2. Flammability Behavior

To further study the synergistic flame-retardation effect of PN-DOPO/Sep@AlPO_4_ compounds on the PEO/PBAT system, the flammability behaviour of the PEO/PBAT/PN-DOPO/Sep@AlPO_4_ composite was evaluated using a cone calorimeter test, and the data are summarised in Table 3. The ignition time (TTI) is usually used to represent the duration before polymer combustion; a high TTI indicates a difficulty to ignite the polymer using a luminous flame [34]. As shown in Table 3, the TTI of samples after incorporating PN-DOPO or PN-DOPO/Sep@AlPO_4_ compounds obviously increased, compared with neat PEO/PBAT. Specifically, it was extended (about 17 s) when 10 wt% PN-DOPO and 5 wt% Sep@AlPO_4_ were introduced into the PEO/PBAT system, and the composite exhibited a TTI of 62 s. The increase in TTI suggested that the PEO/PBAT/PN-DOPO/Sep@AlPO_4_ composites could delay the early thermal decomposition [35].

**Figure 1:**
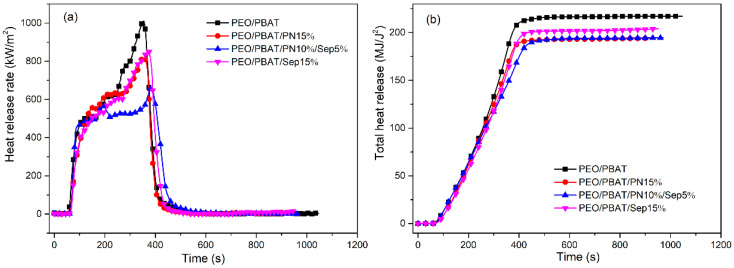
HRR (**a**) and THR (**b**) curves of PEO/PBAT and flame-retardant PEO/PBAT composites.

Figure 1 provides the curves of flame-retarded PEO/PBAT composites including the heat release rate (HRR) and total heat release (THR). As shown in Figure 1, PEO/PBAT burned violently and reached a peak of HRR (p-HRR) of 1021.4 kW/m^2^. With the incorporation of 15 wt% PN-DOPO, the p-HRR of PEO/PBAT/PN15% decreased to 815.7 kW/m^2^. When 15 wt% PN-DOPO was introduced into PEO/PBAT blends, the p-HRR value of the composites reached 851.6 kW/m^2^. After the incorporation of 10 wt% PN-DOPO and 5 wt% Sep@AlPO_4_ into PEO/PBAT, the p-HRR reached only 657.8 kW/m^2^ and decreased by 35.6%. The time to p-HRR (T_PHRR_) was also significantly delayed to 385 s for PEO/PBAT/PN10%/Sep5% compared to that of 354 s for PEO/PBAT. The increase in T_PHRR_ indicated that the PEO/PBAT/PN-DOPO/Sep@AlPO_4_ composites were more difficult to burn and showed delayed thermal decomposition compared to neat PEO/PBAT [7,30]. As depicted in Table 3, the THR and average HRR (av-HRR) of PEO/PBAT were 218.5 MJ/m^2^ and 574.3 kW/m^2^, respectively. After adding PN-DOPO, the THR and aV-HRR values were remarkably decreased. Additionally, the THR and av-HRR values of PEO/PBAT/PN10%/Sep5% decreased by 11.0% and 23.0%, respectively, compared to those of PEO/PBAT.

**Figure 2:**
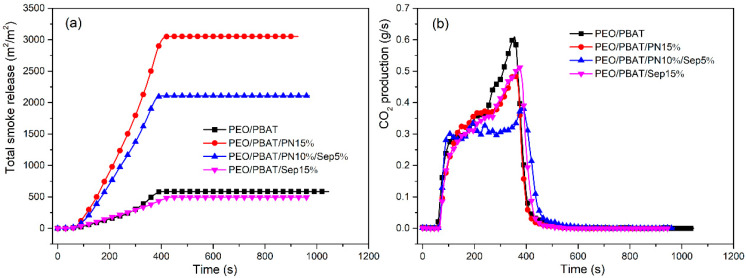
TSR (**a**) and carbon dioxide production (**b**) curves of PEO/PBAT and flame-retardant PEO/PBAT composites.

Figure 2 presents the total smoke release (TSR) and carbon dioxide production curves. As shown in Figure 2, the TSR value of PEO/PBAT was 579.0 m^2^/m^2^, which increased to 3044 m^2^/m^2^ when 15 wt% PN-DOPO was introduced into PEO/PBAT because the composite underwent an incomplete combustion [36]. After the incorporation of 10 wt% PN-DOPO and 5 wt% Sep@AlPO_4_ into PEO/PBAT, the TSR decreased to 2094 m^2^/m^2^. This decrease in TSR may be due to the formation of stable residues, which act as effective physical barriers [25]. It should be noted that the TSR values of PEO/PBAT/PN15% and PEO/PBAT/PN10%/Sep5% are significantly higher than those of PEO/PBAT or PEO/PBAT/Sep15%. Moreover, PEO/PBAT/PN10%/Sep5% exhibited a higher average COY value (0.18 kg/kg) and lower peak CO_2_ production (0.36 g/s) than PEO/PBAT (0.04 kg/kg of COY and 0.62 g/s of CO_2_ production), which might be attributed to the incomplete combustion of flame-retarded composites with the addition of PN-DOPO and Sep@AlPO_4_. The results of TSR, CO_2_ production and av-COY suggest that the incorporation of PN-DOPO/Sep@AlPO_4_ into PEO/PBAT blends results in notable gas-phase flame retardancy and synergistic smoke suppression effects [37].

Based on the abovementioned analysis, the addition of PN-DOPO and Sep@AlPO_4_ could obviously reform the flame retardance of PEO/PBAT blends. Thus, PN-DOPO/Sep@AlPO_4_ displayed remarkable synergistic flame-retardant and smoke suppression effects for flame-retarded PEO/PBAT composites.

### 3.3. Thermal Stability Analysis

**Figure 3:**
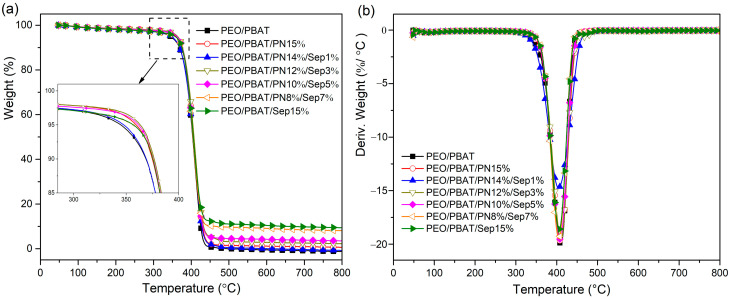
TGA (**a**) and DTG (**b**) curves of PEO/PBAT and flame-retardant PEO/PBAT composites under N_2_ atmosphere.

Figure 3 presents the TGA and differential thermogravimetric analysis (DTG) curves of the flame-retarded composites in nitrogen atmosphere, and the detailed parameters are summarised in Table 4. The corresponding data include T_5%_, which is the temperature of 5 wt% weight loss, and T_max_, which is the maximum decomposition temperature at the maximum mass loss rate.

The thermal decomposition of PEO/PBAT mainly occurred between 300 °C and 450 °C in one single step. The T_5%_ and T_max_ were 344.9 °C and 409.5 °C. The mass loss rapidly increased after increasing the temperature, and the residual char could be ignored at 800℃. The thermal degradation of PEO/PBAT/PN15% composites also exhibited a one-step weight loss, with T_5%_ = 357.5 °C and T_max_ = 409.7 °C. The residual amount increased slightly to 1.86 wt%. After adding Sep@AlPO_4_ to PEO/PBAT, the onset thermal decomposition temperature increased to 356.8 °C, and the final residue was substantially increased to 10.27 wt%. When PN-DOPO was combined with Sep@AlPO_4_ in the PEO/PBAT system, the thermal degradation of the composites also exhibited one-step mass loss, and the TGA and DTG curves of the composites were similar to those for PEO/PBAT.

As listed in Table 4, the apparent increases in T_5%_ indicated that the combination of PN-DOPO and Sep@AlPO4 enhanced the initial thermal stability of PEO/PBAT. Moreover, the char residues of the composites gradually increased with the increase in the amount of Sep@AlPO_4_. For PEO/PBAT/PN12%/Sep3%, the sample displayed the highest T_5%_ and T_max_ (363.4 °C and 410.7 °C, respectively). T_5%_ increased by 18.5 °C compared to that of the PEO/PBAT blend, and the residual amount increased to 3.54 wt%. This enhancement in the initial thermal stability and char yield can be ascribed to the promoting effect of the FRs and sepiolite on the formation of protective layers [38,39]. Thus, the addition of PN-DOPO/Sep@AlPO_4_ into PEO/PBAT may have a synergistic effect on the final residue, which can enhance the thermal stability of composites and delay the emission of the decomposition products. The TG and DTG curves of PEO/PBAT and flame-retarded PEO/PBAT composites under air atmosphere are shown in Figure S1, and the relevant data are listed in Table S1. Similar to the results in nitrogen atmosphere, the char residues of PEO/PBAT/PN-DOPO/Sep@AlPO4 composites were gradually increased by increasing the Sep@AlPO4 contents. The crystallization behavior of the composites was also determined by differential scanning calorimetry (DSC) (Figure S2, Table S2).

### 3.4. TGA-FTIR Analysis

**Figure 4:**
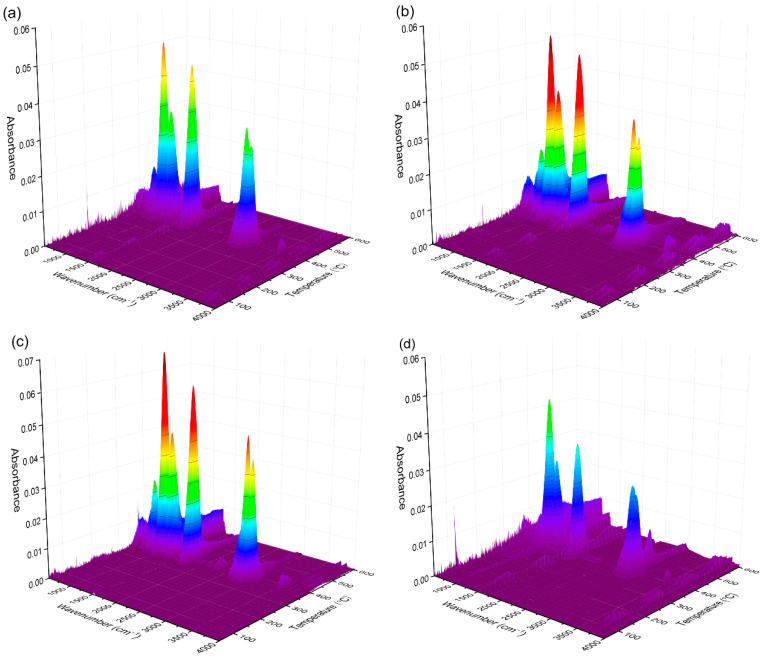
Three-dimensional TGA-FTIR spectra of (**a**) PEO/PBAT, (**b**) PEO/PBAT/PN15%, (**c**) PEO/PBAT/PN10%/Sep5% and (**d**) PEO/PBAT/Sep15%.

Composition analysis of the pyrolysis gas products was conducted through TGA–FTIR testing. Three-dimensional TGA–FTIR spectra of the thermal decomposition of the PEO/PBAT, PEO/PBAT/PN15%, PEO/PBAT/PN10%/Sep5% and PEO/PBAT/Sep15% samples are shown in Figure 4a–d, respectively. Clearly, all samples exhibited similar characteristic bands in 1000–1300 cm^−1^, 1600–1800 cm^−1^ and 2800–3000 cm^−1^, corresponding to ester groups, carbonyl compounds and hydrocarbons, respectively. Figure 4b,c show several new bands compared to the spectra in Figure 4a,d. The new peaks at 700–1400 cm^−1^ could be ascribed to the pyrolysis products of DOPO [25]. Furthermore, the intensity of the peaks of PEO/PBAT and PEO/PBAT/Sep15% was weaker than that of PEO/PBAT/PN10%/Sep5%, indicating that the gas-phase effect of the PEO/PBAT/PN-DOPO/Sep@AlPO_4_ composites was superior to that of PEO/PBAT.

**Figure 5:**
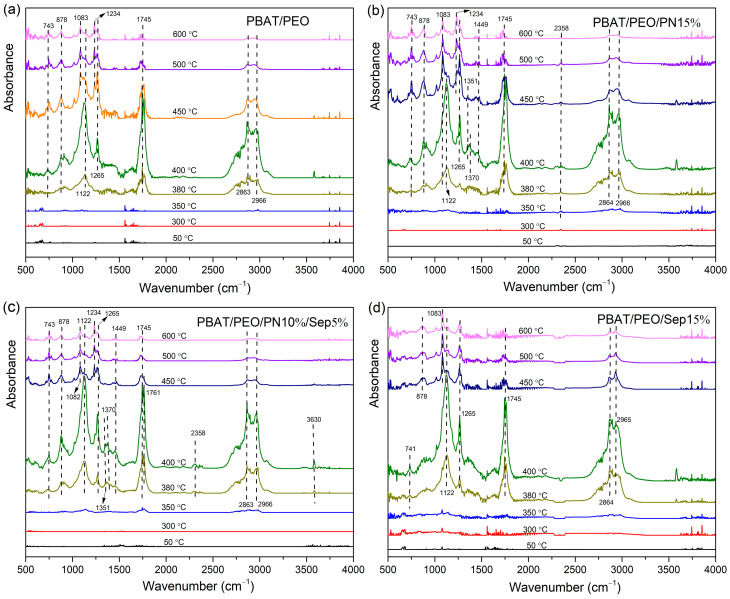
TGA-FTIR spectra of the gaseous phase during the thermal decomposition for (**a**) PEO/PBAT, (**b**) PEO/PBAT/PN15%, (**c**) PEO/PBAT/PN10%/Sep5% and (**d**) PEO/PBAT/ Sep15% at different temperatures.

In order to explore the detailed changes in pyrolysis gas products, the TGA-FTIR spectra of PEO/PBAT, PEO/PBAT/PN15%, PEO/PBAT/PN10%/Sep5% and PEO/PBAT/Sep15% composites at the different decomposition temperature are expressed in Figure 5. For all four samples, the common characteristic absorption peaks, i.e., hydrocarbons (2863 and 2966 cm^−1^), carbonyl compounds (1745 cm^−1^), aliphatic esters (1122, 1234 and 1265 cm^−1^), C–O bonds (1083 cm^−1^) and C–H bonds (743 and 878 cm^−1^) [1,8], occurred at 380–440 °C. Moreover, the peaks at 3500–4000 cm^−1^ were ascribed to water. For the PBAT/PEO/PN15% sample, the peaks occurred at 1351 and 1370 cm^−1^ were ascribed to the –P=O bond. The peak that occurred at 1449 cm^−1^ (corresponding to P–O–C_Ar_) was weak at 400–600 °C [19,38]. The volatiles of the DOPO-based compounds were formed owing to the introduction of PN-DOPO into the PEO/PBAT matrix. When PN-DOPO/Sep@AlPO_4_ was added to PEO/PBAT, absorption peaks appeared at 1351 cm^−1^ and 1370 cm^−1^, assigned to –P=O at 380–450 °C, and at 1449 cm^−1^ corresponding to a P–O–C_Ar_ bond appearing at 380 °C, respectively, compared to the PEO/PBAT and PEO/PBAT/Sep15% revealed in Figure 5a,d. These results indicated that the introduction of Sep@AlPO_4_ alone could not effectively increase the gas-phase products. After adding the combination of PN-DOPO and Sep@AlPO_4_, the blends exhibited absorption peaks of phosphorous-containing groups and an increase in gas-phase volatiles during the thermal degradation process, suggesting that the flame retardancy of the PEO/PBAT/PN-DOPO/Sep@AlPO_4_ composites were improved through a gas-phase flame-retardant mechanism. 

### 3.5. Condensed-Phase Analysis

#### 3.5.1. Morphologies of Residues

**Figure 6:**
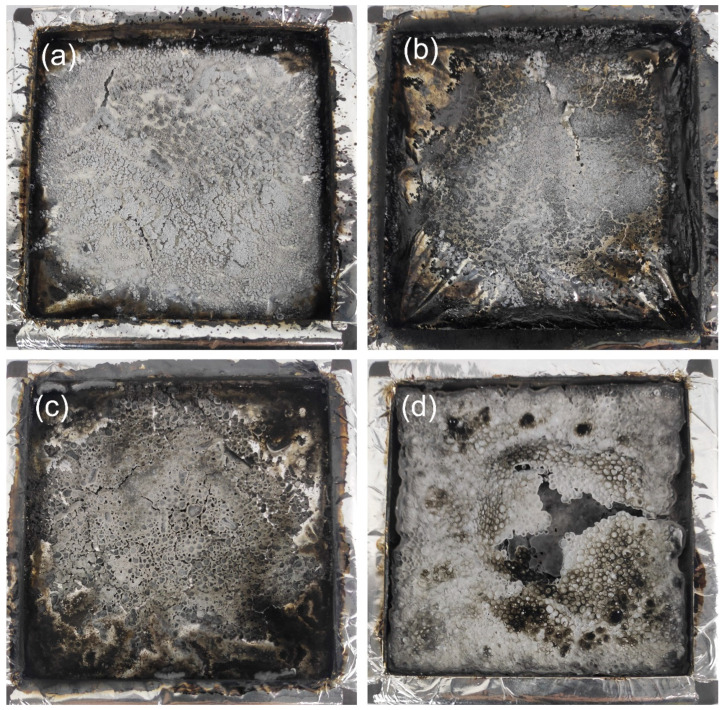
Photographs of the char residues for (**a**) PEO/PBAT, (**b**) PEO/PBAT/PN15%, (**c**) PEO/PBAT/PN10%/Sep5% and (**d**) PEO/PBAT/Sep15%.

The morphologies of the char residues were investigated to examine the specific flame-retardant mechanism of PEO/PBAT/PN-DOPO/Sep@AlPO_4_ composites in the condensed phase. The digital photos of the residues are revealed in Figure 6.

Figure 6a indicates that the char residues left behind after combustion of the PEO/PBAT matrix exhibited a discontinuous and loose structure, with some cracks and poor quality. After incorporating 15 wt% PN-DOPO, the blends represented a very thin and incomplete char layer after burning, as depicted in Figure 6b. This form of residue could not protect the substrate from combustion or isolate the exchange of the combustion gases. In the cases of PEO/PBAT/PN10%/Sep5% and PEO/PBAT/Sep15%, the morphologies of the residues are substantially different from those of PEO/PBAT and PEO/PBAT/PN15% (as in Figure 6c,d). Especially in the case of the PEO/PBAT/PN10%/Sep5% composites, a full, thick and dense residual carbon layer was observed after the combustion (Figure 6c). The results indicated that combining PN-DOPO and Sep@AlPO_4_ could enhance the quality of the residues and contribute to the flame-retardant effect in the condensed phase.

**Figure 7:**
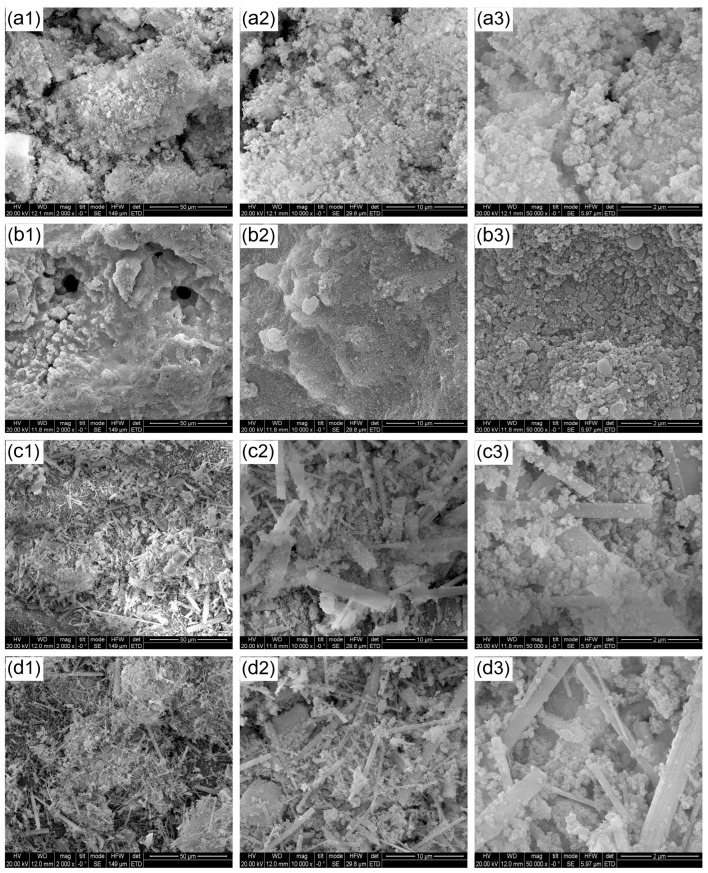
SEM images of the residues on PEO/PBAT ((**a1**)—2000×; (**a2**)—10,000×; (**a3**)—50,000×), PEO/PBAT/PN15% ((**b1**)—2000×; (**b2**)—10,000×; (**b3**)—50,000×), PEO/PBAT/PN10%/Sep5% ((**c1**)—2000×; (**c2**)—10,000×; (**c3**)—50,000×) and PEO/PBAT/Sep15% ((**d1**)—2000×; (**d2**)—10,000×; (**d3**)—50,000×).

The microscopic morphologies of the residues of the flame-retarded PEO/PBAT composites after the cone calorimetry test were further observed using SEM. The residue surface of the pure PEO/PBAT matrix presented a relatively loose and inhomogeneous structure, as in Figure 7(a1–a3), including some crevasses because of insufficient char formation during the burning process. As for PEO/PBAT/PN15%, uniform and dense char residues with a few holes after combustion can be found in Figure 7(b1–b3). With regard to PEO/PBAT/PN10%/Sep5% and PEO/PBAT/Sep15%, the morphologies of the char layer were markedly different from those of PEO/PBAT and PEO/PBAT/PN15%, as shown in Figure 7(c1–c3,d1–d3), presenting a more continuous and compact fibre-like structure. The results suggested that the addition of Sep@AlPO_4_ increased the carbon yield and promoted the formation of phosphorus-rich residues, making a denser char layer [38,40]. The layer exhibited higher thermal stability and strength, which could effectively prevent the escape of flammable volatiles and heat transfer during burning, further improving the flame retardance of the PEO/PBAT blends.

#### 3.5.2. Chemical Compositions of the Residues

The elemental compositions of the residues were characterised using SEM–EDS, and the results are summarised in Table 5. In the case of the PEO/PBAT sample, only carbon and oxygen elements were detected. In addition to carbon and oxygen, phosphorus (19.1 wt%) was detected in the residue of the PEO/PBAT/PN15% composites. The results showed that there were phosphorus-containing products in the residue after the incorporation of DOPO-based flame retardants, while the oxygen, aluminium, silicon, magnesium elements and a relatively lower carbon content (11.7 wt%) and phosphorus content (12.8 wt%) were detected on the char layer of PEO/PBAT/Sep15% sample. These elements were the main components of aluminium phosphates and sepiolite residues [41,42]. After adding PN-DOPO/Sep@AlPO_4_ into the PEO/PBAT blends, the sepiolite components (silicon, magnesium and aluminium) were observed in the char residues along with an increase in the phosphorus content (21.2 wt%). This further illustrates that PN-DOPO and Sep@AlPO_4_ can catalyse the reactions to form phosphorus- and oxide-rich residues, which exhibit barrier effects on oxygen and heat transfer.

In summary, PN-DOPO and Sep@AlPO_4_ were added to the PEO/PBAT blends, resulting in the composites exhibiting an increase in gas-phase volatiles. Additionally, absorption peaks of phosphorous-containing products were observed during combustion. The hybrid compounds rich in phosphorus, silicon and aluminium oxides can form a continuous and dense char layer. In this case, the flame retardance and thermal stability of the PEO/PBAT blends were considerably improved. Therefore, the PEO/PBAT/PN-DOPO/Sep@AlPO_4_ composites exhibited a synergistic flame-retardant effect through gas-phase quenching and char formation in the condensed phase.

### 3.6. Rheological Behavior

**Figure 8:**
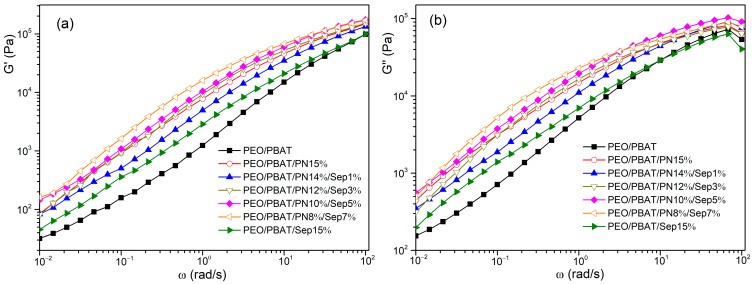
Storage modulus (**a**) and loss modulus (**b**) of PEO/PBAT and flame-retardant PEO/PBAT composites.

Rheological measurements have recently been used to study the combustion behaviour and fire resistance of polymeric composites. The effects of melt flow characteristics on flame-retarded composites were studied [24,43,44]. The influence of incorporating PN-DOPO/Sep@AlPO_4_ into the PEO/PBAT blends on their rheological properties was examined. Furthermore, the relation of the storage modulus (G′) and loss modulus (G″) of PEO/PBAT and flame-retardant PEO/PBAT composites with frequency (ω) at 150 °C are illustrated in Figure 8, respectively. The G′ and G″ values of the PEO/PBAT/PN-DOPO/Sep@AlPO_4_ composites were higher than those of the PEO/PBAT and PEO/PBAT/Sep@AlPO_4_ blends over the whole frequency range. The increased G′ and G″ values of PEO/PBAT/PN-DOPO/Sep@AlPO_4_ might be attributed to interfacial interactions, and both the DOPO-based FRs and sepiolite could affect the viscoelastic behaviour and relaxation of the PEO/PBAT matrix [45,46]. Among these samples, PEO/PBAT/PN8%/Sep7% exhibited the highest G′ and G″ values, indicating that the interfacial interaction between the PN-DOPO and PEO/PBAT matrices was improved via the immobilisation of Sep@AlPO_4_ [44].

**Figure 9:**
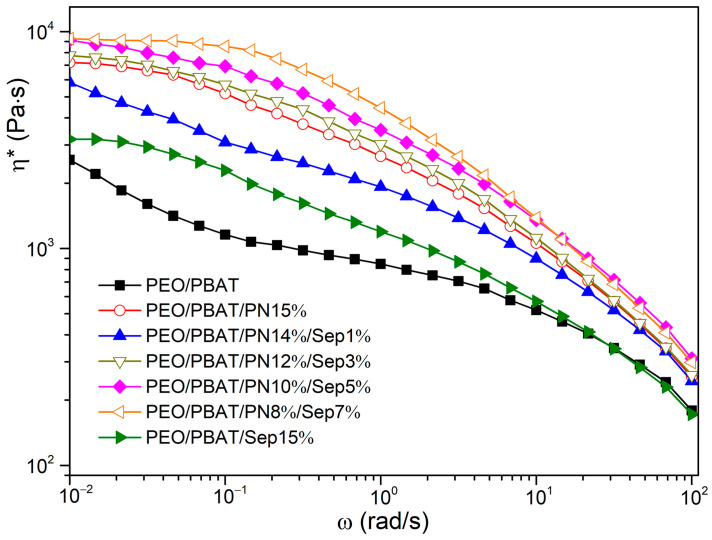
Complex viscosity of PEO/PBAT and flame-retardant PEO/PBAT composites.

The complex viscosity (η*) of PEO/PBAT and flame-retardant PEO/PBAT is shown in Figure 9. The η* value increased with increasing Sep@AlPO_4_ content for the PEO/PBAT/PN-DOPO/Sep@AlPO_4_ system. The η* values of the PEO/PBAT/PN-DOPO/Sep@AlPO_4_ composites were obviously larger than the those of the PEO/PBAT and PEO/PBAT/Sep@AlPO_4_ blends, which might be due to the network structure formed by FRs and inorganic particles [47]. The PEO/PBAT/PN8%/Sep7% sample had the highest η* value. The flame-retarded PEO/PBAT/Sep@AlPO_4_ composites had high viscosity in the melted state, which could suppress the volatilisation of decomposition products and limit the flow of molecular chains during combustion [24]. High viscosity or storage modulus can also promote the crosslinking of condensed phases, and similar experimental results were reported by Li et al. [47] and He et al. [48]. According to Seraji et al. [49], the interfacial between the FR and polymer was improved with the increase in loading and the platform at the low-frequency region was more prominent, indicating the crosslinking network structure changed from from liquid-like to solid-like behaviour. The results indicate that a three-dimensional network is formed for flame-retardant composites, which is helpful to form a dense layer to enhance the fire resistance of composites during combustion. Therefore, high viscosity and modulus could promote the cross-linking of condensed phases during char formation, thus improving the flame retardance of the composites.

### 3.7. Mechanical Properties

**Figure 10:**
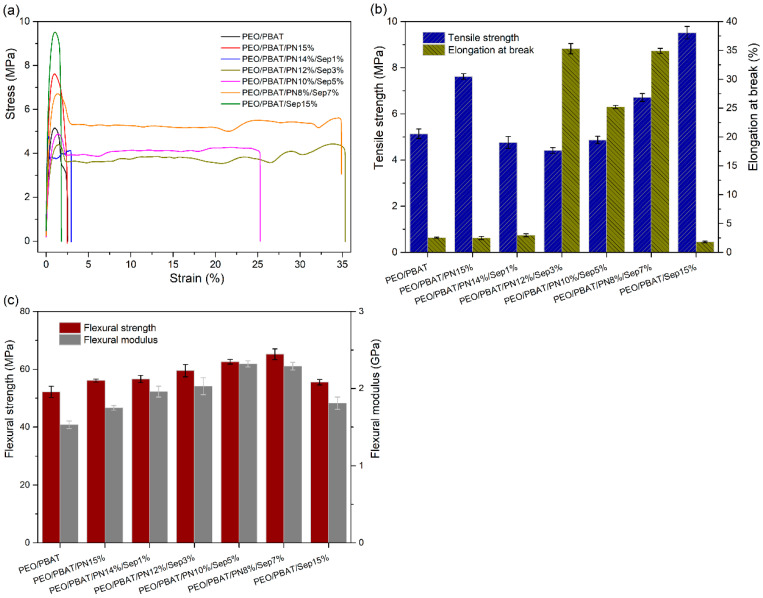
Mechanical properties of PEO/PBAT and flame-retarded PEO/PBAT composites: (**a**) stress–strain curves, (**b**) tensile strength and elongation at break and (**c**) flexural strength as well as modulus.

The effects of PN-DOPO combined with Sep@AlPO_4_ on the mechanical performances of the PEO/PBAT system were also investigated. The stress–strain curves and tensile strength, elongation at break, flexural strength as well as modulus of all samples are shown in Figure 10. The tensile strength, flexural strength, flexural modulus and elongation at break of unfilled PEO/PBAT were 5.14 ± 0.21 MPa, 52.2 ± 1.94 MPa, 1.53 ± 0.05 GPa and 2.54 ± 0.09%, respectively. At the addition of 15 wt% PN-DOPO, the tensile strength and flexural modulus of the sample increased by 48.2% and 14.4%, respectively, whereas the elongation at break remained almost unchanged. Incorporating PN-DOPO and Sep@AlPO_4_ into the composites had a positive effect on the mechanical performances of pure PEO/PBAT. The flexural properties and elongation at break of the PEO/PBAT/PN-DOPO/Sep@AlPO_4_ composites exhibited a clear increase compared with those of PEO/PBAT. After adding 10 wt% PN-DOPO and 5 wt% Sep@AlPO_4_, the flexural strength, flexural modulus and elongation at break of the PEO/PBAT/PN10%/Sep5% sample were increased by 19.9%, 51.6% and 892%, respectively, compared with neat PEO/PBAT.

The improvement in the flexural properties and elongation at break of flame-retarded PEO/PBAT may be related to the dispersion of PN-DOPO and Sep@AlPO_4_ in the matrix during the melting process [24,50]. Moreover, the elongation at break increased with the increase in the amount of Sep@AlPO_4_, suggesting that PN-DOPO/Sep@AlPO_4_ compounds effectively improved the toughness of the matrix. Herein, the multi-dimensional particle structure formed a good interface with the matrix, which was beneficial for the transfer from the polymer chain to the particles [51,52]. Thus, the PEO/PBAT/PN-DOPO/Sep@AlPO_4_ composites exhibited better mechanical properties.

**Figure 11:**
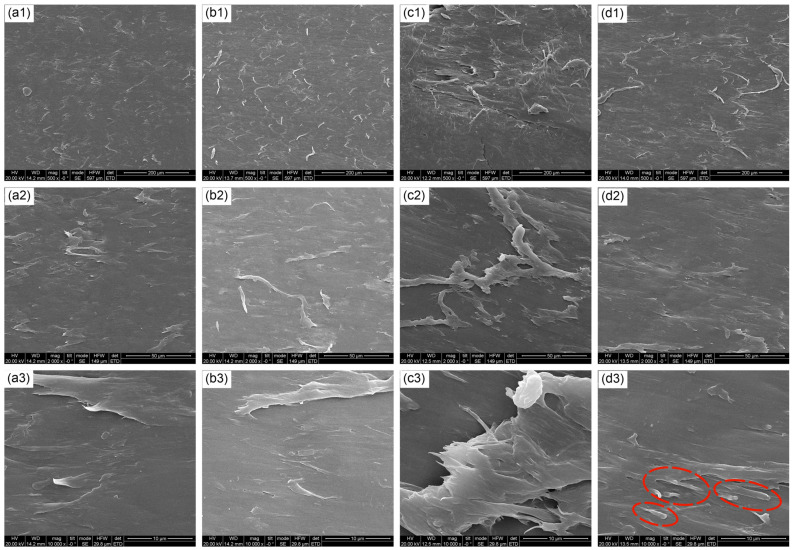
SEM images of fractured surfaces for PEO/PBAT ((**a1**)—500×; (**a2**)—2000×; (**a3**)—10,000×), PEO/PBAT/PN15% ((**b1**)—500×; (**b2**)—2000×; (**b3**)—10,000×), PEO/PBAT/PN10%/Sep5% ((**c1**)—500×; (**c2**)—2000×; (**c3**)—10,000×) and PEO/PBAT/Sep15% ((**d1**)—500×; (**d2**)—2000×; (**d3**)—1000×) at different magnifications.

To confirm the improvement in mechanical properties, SEM was used to observe the fracture surface of the PEO/PBAT and flame-retarded PEO/PBAT composites. As depicted in Figure 11(a1–a3), PEO/PBAT exhibited a flat and smooth surface, characteristic of the typical brittle fracture of thermoplastic resin, suggesting that plastic deformation did not occur during the fracture process [52,53]. PEO/PBAT/PN15% exhibited similar cross-sectional characteristics to pure PEO/PBAT. In contrast, PEO/PBAT/PN8%/Sep7% exhibited a rough surface with protrusions and pulling-out phenomena (Figure 11(c1–c3)), which may be due to the interface interaction between the fillers and the matrix. These obvious protrusions played an important role in hindering crack propagation [54]. Additionally, the improvement in the surface roughness confirmed the main fracture mode of plastic deformation, resulting in a considerable increase in the elongation at break of the PEO/PBAT/PN-DOPO/Sep@AlPO_4_ composites. Moreover, PEO/PBAT/Sep15% also presented a rough surface with obvious protrusions and orientations, as shown in the high-magnification SEM image (Figure 11(d3)), originating from the inorganic properties of fibrous sepiolite (marked with a red ellipse) [55]. Hence, the PEO/PBAT/Sep15% sample exhibited the maximum tensile strength. 

In summary, when PN-DOPO and modified sepiolite were introduced into the PEO/PBAT system, the composites exhibited considerable synergistic flame-retardant effects on combustion behaviour, thermal stability, melt viscosity and carbonisation. Moreover, compared with the PEO/PBAT, PEO/PBAT/PN-DOPO and PEO/PBAT/Sep@AlPO_4_ systems, the PEO/PBAT/PN-DOPO/Sep@AlPO_4_ composites showed improved mechanical performances, such as elongation at break and flexural performance, exhibiting cross-sectional plastic deformation characteristics.

## 4. Conclusions

In this study, a DOPO-based flame retardant (PN-DOPO) and aluminium phosphate-coated sepiolite (Sep@AlPO_4_) were jointly used to improve the flame retardance and mechanical performances of a PEO/PBAT system through melt mixing. The influences of PN-DOPO/Sep@AlPO_4_ mixtures on the flame retardance, thermal decomposition, carbonisation and mechanical performances of PEO/PBAT/PN-DOPO/Sep@AlPO_4_ composites were systemically investigated using various analytical instruments. After adding 10 wt% PN-DOPO and 5 wt % Sep@AlPO_4_, the LOI value of the composites raised to 23.7% and achieved a V-1 rating. The p-HRR, THR and av-HRR values of PEO/PBAT/PN10%/Sep5% decreased by 35.6%, 11.0% and 23.0%, respectively, compared with those of PEO/PBAT. Furthermore, the TGA results elucidated that PN-DOPO/Sep@AlPO_4_ could improve the initial thermal stability and char yield of the PEO/PBAT system. In the TGA–FTIR spectra, the PEO/PBAT/PN-DOPO/Sep@AlPO_4_ blends exhibited absorption peaks characteristic of phosphorous-containing groups. They also exhibited an increase in the gas-phase volatiles during their thermal decomposition process. The results of the SEM–EDS test confirmed that the PN-DOPO/Sep@AlPO_4_ combination could promote a dense carbon layer rich in phosphorus, silicon and aluminium oxides in the condensed phase. Moreover, the PEO/PBAT/PN-DOPO/Sep@AlPO_4_ composites exhibited higher flexural properties and elongation at break than other samples. Thus, combining Sep@AlPO_4_ with PN-DOPO and introducing them into PEO/PBAT composites presented an evident synergistic flame-retardant effect. Moreover, Sep@AlPO_4_ is a promising and effective synergistic agent that can simultaneously improve the flame retardance, thermal stability and mechanical performances of flame-retarded PEO/PBAT blends.

## Figures and Tables

**Table 1:** Composition of the PEO/PBAT/PN-DOPO/Sep@AlPO_4_ composites.

Samples	PEO (wt%)	PBAT (wt%)	PN-DOPO (wt%)	Sep@AlPO_4_ (wt%)
PEO/PBAT	60	40	0	0
PEO/PBAT/PN15%	60	40	15	0
PEO/PBAT/PN14%/Sep1%	60	40	14	1
PEO/PBAT/PN12%/Sep3%	60	40	12	3
PEO/PBAT/PN10%/Sep5%	60	40	10	5
PEO/PBAT/PN8%/Sep7%	60	40	8	7
PEO/PBAT/Sep15%	60	40	0	15

**Table 2:** The UL-94 and LOI tests of PEO/PBAT and PEO/PBAT/PN-DOPO/Sep@AlPO_4_ composites.

Samples	UL-94 (3.2 mm)				LOI (%)
	t_1_ (s)	t_2_ (s)	Dripping	Rating	
PEO/PBAT	>30	>30	Yes	No Rating	20.2 ± 0.1
PEO/PBAT/PN15%	19.6	5.1	Yes	V-2	22.6 ± 0.1
PEO/PBAT/PN14%/Sep1%	18.5	3.4	Yes	V-2	22.8 ± 0.2
PEO/PBAT/PN12%/Sep3%	12.7	4.6	No	V-1	23.2 ± 0.2
PEO/PBAT/PN10%/Sep5%	11.4	2.8	No	V-1	23.7 ± 0.2
PEO/PBAT/PN8%/Sep7%	16.8	6.2	Yes	V-2	23.1 ± 0.3
PEO/PBAT/Sep15%	20.1	5.5	Yes	V-2	21.8 ± 0.2

**Table 3:** Cone calorimetric results for the PEO/PBAT/PN-DOPO/Sep@AlPO_4_ composites.

Samples	TTI	p-HRR	av-HRR	T_PHRR_	THR	TSR	av-COY
	(s)	(kW/m^2^)	(kW/m^2^)	(s)	(MJ/m^2^)	(m^2^/m^2^)	(kg/kg)
PEO/PBAT	55	1021.4	574.3	354	218.5	579.0	0.04
PEO/PBAT/PN15%	61	815.7	486.0	359	194.2	3044.3	0.22
PEO/PBAT/PN10%/Sep5%	62	657.8	442.3	385	194.5	2094.1	0.18
PEO/PBAT/Sep15%	59	851.6	501.7	373	204.0	483.9	0.05

**Table 4:** TGA and DTG data of PEO/PBAT and flame-retardant PEO/PBAT composites.

Samples	T_5%_ (°C)	T_max_ (°C)	Residues at 800 °C (wt%)
PEO/PBAT	344.9	409.5	0.06
PEO/PBAT/PN15%	357.5	409.7	1.86
PEO/PBAT/PN14%/Sep1%	347.7	407.2	0.62
PEO/PBAT/PN12%/Sep3%	363.4	410.7	3.54
PEO/PBAT/PN10%/Sep5%	361.8	408.1	4.76
PEO/PBAT/PN8%/Sep7%	357.0	406.4	9.18
PEO/PBAT/Sep15%	356.8	407.6	10.27

**Table 5:** Element contents of the char residues of PEO/PBAT and flame-retarded PEO/PBAT composites obtained via EDS.

Samples	Elemental Content (wt%)					
	C	O	P	Al	Si	Mg
PEO/PBAT	18.3	81.7	-	-	-	-
PEO/PBAT/PN15%	31.1	49.8	19.1	-	-	-
PEO/PBAT/PN10%/Sep5%	12.9	38.6	21.2	4.5	16.6	6.2
PEO/PBAT/Sep15%	11.7	37.5	12.8	5.5	23.1	9.4

## Data Availability

The data presented in this study are available on request from the corresponding author.

## Supplementary Materials

The following supporting information can be downloaded at: https://www.mdpi.com/article/10.3390/polym16010045/s1, Figure S1: TGA (a) and DTG (b) curves of flame-retarded PEO/PBAT composites in air atmosphere; Figure S2: DSC curves of PEO/PBAT and flame-retarded PEO/PBAT composites in nitrogen atmosphere, (a) melting curves and (b) crystallization curves; Table S1: TGA and DTG data of flame-retarded PEO/PBAT composites in air atmosphere; Table S2: DSC data of PEO/PBAT and flame-retarded PEO/PBAT composites.
