# Supplementary material for: Synergistic Effects of 9,10-Dihydro-9-oxa-10-phosphaphenanthrene-10-oxide-Based Derivative and Modified Sepiolite on Flame-Retarded Poly (Ethylene Oxide)–Poly (Butylene Adipate-Co-Terephthalate) Composites
Source: Polymers (Basel). 2023 Dec 22;16(1):45. doi: 10.3390/polym16010045 (PMC10781121; doi:10.3390/polym16010045)
Supplement: Supplementary file 1 [file polymers-16-00045-s001.zip › polymers-2735973-supplementary.pdf]

Supplementary Information:

The TG and DTG curves of PEO/PBAT and flame-retarded PEO/PBAT composites under air atmosphere are shown in Figure S1, and the relevant data are listed in Table S1. PEO/PBAT and its composites have different TG and DTG curves under air and nitrogen atmospheres. There are two major stages of weight loss under air atmospheres of pure PEO/PBAT and PEO/PBAT composites, while only one stage is observed under N_2 atmosphere. The two stages were mainly the weight loss of the matrix, and the similar results were reported by Yu et al. [Chen, Z.W.; Yu, Y.; Zhang, Q.W.; et al. Preparation of phosphorylated chitosan-coated carbon microspheres as flame retardant and its application in unsaturated polyester resin. *Polym. Advan. Technol.* **2019**, *30*, 1933–1942.] and Chen et al. [Chen, X.L.; Wang, K.; Li, S.X.; et al. Effects of flame retardants integrated with citrate and ammonium polyphosphate on thermal stability and flame retardancy of thermoplastic polyurethane elastomer. *Polym. Advan. Technol.* **2021**, *32*, 2866–2878.]. Pure PEO/PBAT leaves only 1.38 wt% residues left at 800 °C, indicating that the matrix has poor carbon-forming ability. Similar to the results in nitrogen atmosphere, the char residues of PEO/PBAT/PN-DOPO/Sep@$AlPO_4$ composites were gradually increased by increasing the Sep@$AlPO_4$ contents. When the amount of Sep@$AlPO_4$ was 7 wt%, the T_{max} and residues of PEO/PBAT/PN8%/Sep7% were the highest, reaching 362.5 °C and 10.76 wt%, respectively. The high yield of residues of the composites suggests that the combination of PN-DOPO and Sep@$AlPO_4$ into PEO/PBAT blends may have a synergistic effect on the final residue.

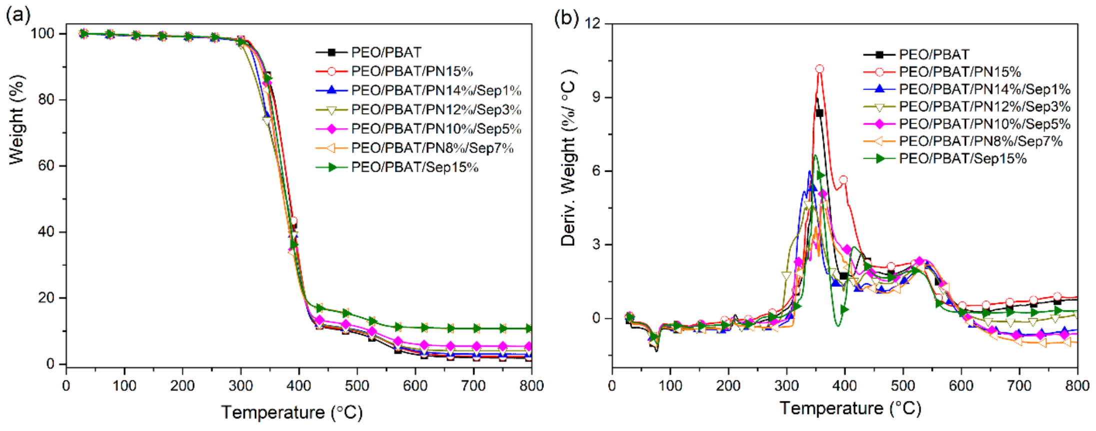

Figure S1. TGA (a) and DTG (b) curves of flame-retarded PEO/PBAT composites in air atmosphere.

Table S1. TGA and DTG data of flame-retarded PEO/PBAT composites in air atmosphere.

Samples	$T_{5\%}$ (°C)	T_{max} (°C)	The residues at 800 °C (wt%)
PEO/PBAT	322.7	352.5	1.38
PEO/PBAT/PN15%	325.6	356.3	2.26
PEO/PBAT/PN14%/Sep1%	320.1	340.1	3.02
PEO/PBAT/PN12%/Sep3%	316.2	346.2	4.05
PEO/PBAT/PN10%/Sep5%	322.5	357.1	5.45
PEO/PBAT/PN8%/Sep7%	321.8	362.5	10.76
PEO/PBAT/Sep15%	325.5	349.3	10.81

The crystallization behavior of PEO/PBAT mixture was determined by differential scanning calorimetry (DSC). The DSC plots in the N_2 atmosphere are presented in Figure S2, and the experimental results are listed in Table S2. There was only one exothermic peak or endothermic peak corresponding to the sample in each DSC curve. The crystallization temperature (T_c) and the melting peak temperature (T_m) of pure PEO/PBAT were 40.5 °C and 63.2 °C, respectively. The T_c and T_m of PEO/PBAT/PN-DOPO/Sep@$AlPO_4$ composites were gradually increased by the increasing content of Sep@$AlPO_4$. When the amount of Sep@$AlPO_4$ was 7 wt%, the T_c and T_m of PEO/PBAT/PN8%/Sep7% were the highest. When using both PN-DOPO and Sep@$AlPO_4$, the flame-retardant system little influence on the position of the melting peak T_m. These were rather similar to the melt behaviors of the neat PEO/PBAT. Compared with the pure matrix, the decrease in crystallization peak temperature of the composites can be attributed to the heterogeneous nucleation effect of DOPO-based flame retardants and inorganic particles on the matrix [Jia, L.; Zhang W.C.; Tong, B.; Yang, R.J. Crystallization, mechanical and flame-retardant properties of poly (lactic acid) composites with DOPO and DOPO-POSS. *Chinese J. Polym. Sci.* **2018**, *36*, 871–879], [Wang, P.; Chen, L.; Xiao, H. Flame retardant effect and mechanism of a novel DOPO based tetrazole derivative on epoxy resin. *J. Anal. Appl. Pyrol.* **2019**,*139*, 104–113]. In summary, DOPO-based flame retardants are like plasticizer in the polymer, while organic coated sepiolite acts like a filler in the matrix. Therefore, they have different effects on the crystallization and melting behaviors of the PEO/PBAT matrix.

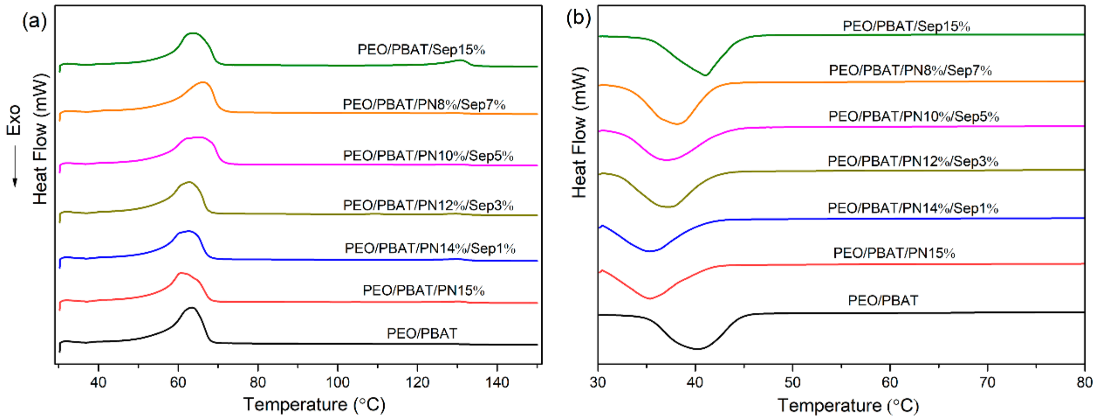

Figure S2. DSC curves of PEO/PBAT and flame-retarded PEO/PBAT composites in nitrogen atmosphere, (a) melting curves and (b) crystallization curves.

Table S2. DSC data of PEO/PBAT and flame-retarded PEO/PBAT composites.

Samples	T_m (°C)	T_c (°C)
PEO/PBAT	63.2	40.5
PEO/PBAT/PN15%	60.6	35.3
PEO/PBAT/PN14%/Sep1%	61.9	36.2
PEO/PBAT/PN12%/Sep3%	62.8	37.1
PEO/PBAT/PN10%/Sep5%	65.3	38.2
PEO/PBAT/PN8%/Sep7%	66.4	39.6
PEO/PBAT/Sep15%	63.9	41.1
